# Foodborne concerns of *Blastocystis* spp. in marine animals (fish, bivalves, and sponges): A systematic review and meta-analysis of global prevalence and subtypes distribution

**DOI:** 10.1016/j.fawpar.2024.e00242

**Published:** 2024-08-25

**Authors:** Mohammad Ghafari-Cherati, Amin Karampour, Seyedeh-Sara Nazem-Sadati, Ali Asghari

**Affiliations:** a Metabolic Diseases Research Center, Research Institute for Prevention of Non-Communicable Diseases, Qazvin University of Medical Sciences, Qazvin, Iran; b Medical Microbiology Research Center, Qazvin University of Medical Sciences, Qazvin, Iran; c Social Determinants of Health Research Center, Research Institute for Prevention of Non-Communicable Diseases, Qazvin University of Medical Sciences, Qazvin, Iran

**Keywords:** *Blastocystis* spp., Prevalence, Subtypes, Marine animals, Systematic review

## Abstract

*Blastocystis* spp. is a common intestinal parasite found in humans and various animals, including marine species like fish, bivalves, and sponges. While traditionally considered non-pathogenic, emerging evidence suggests potential foodborne concerns, especially for vulnerable populations. The present systematic review and meta-analysis reviewed four electronic databases (PubMed, Scopus, Google Scholar, and Web of Science) until June 13, 2024, for studies reporting the prevalence and subtypes (STs) distribution of *Blastocystis* spp. in marine animals, including fish, bivalves, and sponges, to assess foodborne concern and zoonotic importance. In the analysis of 11 studies involving 1329 marine animals from nine countries, five studies/datasets (742 samples) focused on fish, five studies/datasets (567 samples) on bivalves, and one (20 samples) on sponges. This review found that 12.4 % (95 % CI: 4.3–31 %) of marine animals globally were infected by *Blastocystis* spp., with bivalves showing the highest infection rate at 32 % (95 % CI: 13–59.7 %), exceeding sponges with a single study at 10 % (95 % CI: 2.5–32.4 %), and fish at 4.4 % (95 % CI: 2–9.3 %). Sensitivity analysis assessed changes in weighted prevalence after excluding certain studies. A subgroup analysis of *Blastocystis* spp. prevalence was conducted based on publication years, countries, continents, WHO regions, and sample sizes. The data collected indicated that marine animals serve as suitable reservoirs for various *Blastocystis* spp. STs (ST1-ST4, ST7, ST8, ST10, ST14, ST23, ST26, and ST44), with most (except for ST26 and ST44) having the potential for zoonotic transmission. Overall, the findings emphasize the potential for foodborne risk posed by *Blastocystis* spp. in marine animals and highlight the need for improved monitoring and control measures to ensure food safety.

## Highlights

• This review found that 12.4 % (95 % CI: 4.3–31 %) of marine animals globally were infected by *Blastocystis* spp.
• Bivalves had the highest infection rate at 32 % (95 % CI: 13–59.7 %).
• The findings emphasize the potential for foodborne risk posed by *Blastocystis* spp. in marine animals.

## 1 Introduction

*Blastocystis* spp. is a prevalent protozoan parasite found in the digestive tracts of animals and humans globally (Asghari et al., 2024c; Guilavogui et al., 2022). The frequency of *Blastocystis* spp. differs between developed and developing countries, with higher rates usually observed in regions with inadequate sanitation and restricted access to safe water (Asghari et al., 2024b; Mohammad et al., 2018). Risk factors for contracting *Blastocystis* spp. include poor hygiene practices, close contact with infected individuals, and consuming contaminated food or water (Asghari et al., 2024a; Mohammad et al., 2017). Clinical symptoms of infection can vary from mild gastrointestinal discomfort to more severe symptoms like diarrhea, nausea, and abdominal pain (Salvador et al., 2016). The pathogenesis of *Blastocystis* spp. infection is not fully understood, but *Blastocystis* spp. has the ability to regulate the gut microbiome and immune responses to maintain the homoeostasis as well (Roberts et al., 2014; Shams et al., 2024; Vassalos et al., 2008).

The SSU-rRNA gene polymorphism identified 40–44 genetically different variants, or subtypes (STs). 17 have been identified in humans and animals (ST1-ST10, ST12-ST14, ST16, ST23, ST35, and ST41), with ST1–ST4 comprising >90 % of human isolates. ST1–ST4 infections are usually transmitted between humans, while other STs are prevalent among specific host groups such as mammals or birds, spreading through human–animal interactions (Bastaminejad et al., 2024; Santin et al., 2024).

Given the zoonotic significance and genetic variety of this parasitic protozoan, numerous original and/or review studies have been carried out on humans and various animal categories, particularly zoo animals, domestic animals, and pets (Abedi et al., 2022; Asghari et al., 2021b; Asghari et al., 2021a; Badparva et al., 2017; Barati et al., 2022; Fusaro et al., 2024; Kumarasamy et al., 2023; Rostami et al., 2017; Shams et al., 2021, Shams et al., 2022b; Shams et al., 2022a; dos Zanetti et al., 2020). However, there are few studies on the frequency and STs distribution of *Blastocystis* spp. in aquatic animals, possibly because of the challenge in sampling these animals. As many individuals worldwide are beginning to consume aquatic foods like fish and bivalves, the examination for parasitic infections such as *Blastocystis* spp. in these animals is crucial. Thus, this study was conducted to determine the prevalence and STs distribution of *Blastocystis* spp. in marine animals (fish, bivalves, and sponges) using existing information in this area.

## 2 Methods

### 2.1 Ethics approval and study type

The study received approval from the Ethics Committee of Qazvin University of Medical Sciences, Qazvin, Iran (approval no. IR.QUMS.REC.1403.176). It was a global systematic review and meta-analysis for the prevalence and subtype distribution of *Blastocystis* spp. in marine animals (fish, bivalves, and sponges). The research adhered to the PRISMA (Preferred Reporting Items for Systematic Reviews and Meta-Analysis) guidelines (Moher et al., 2015).

### 2.2 Database search

The study analysed four global databases: Medline/PubMed, ProQuest, Scopus, and the Web of Knowledge, for articles published until June 13, 2024. Google Scholar was consulted for grey literature. The search was performed using Medical Subject Heading (MeSH) terms alone or in combination: (“Intestinal Parasites” OR “Parasitic Infections” OR “*Blastocystis* spp.”) AND (“Prevalence” OR “Epidemiology” OR “Frequency” OR “Occurrence”) AND (“Subtype” OR “Subtyping”) AND (“Aquatic animals” OR “Marine animals” OR “Fish” OR “Molluscs” OR “Bivalves” OR “Sponges”). To include more pertinent papers, extra keywords were utilized, and the references of relevant papers were examined. The data collected was input into EndNote X9 software, and duplicate articles were automatically removed. Two researchers independently reviewed the articles.

### 2.3 Inclusion/exclusion criteria

Cross-sectional studies from all languages, regions, and time periods that reported *Blastocystis* spp. prevalence in marine animals using microscopy, molecular, and serological methods were evaluated in this review. Studies on non-marine animals, humans and plants, case reports, commentaries, reviews, and studies that lacked total sample size or *Blastocystis* spp. prevalence rate were excluded from this review.

### 2.4 Data selection criteria

Papers were evaluated for inclusion or exclusion based on the Joanna Briggs Critical Appraisal Checklist for Studies Reporting Prevalence Data (Munn et al., 2014). Studies scoring 4–6 points were considered as moderate-quality, while those scoring 7 points or more were classified as high-quality. Articles with scores ≤3 were excluded. Two researchers extracted key data from the chosen papers, which were then validated by other researchers. The extracted information included the primary author's last name, animal type, quality assessment score, publication year, implementation year, continent, country, World Health Organization (WHO) classification, total sample size, and number of infected samples.

### 2.5 Statistical analysis

Statistical analyses utilized the Comprehensive Meta-Analysis (CMA) v3 software. *P*-values below 0.05 were deemed statistically significant. The random-effects model was employed to evaluate *Blastocystis* spp. prevalence by estimating pooled prevalence and 95 % CIs. Subgroup analysis was conducted to assess the weighted prevalence of infection by animal types, WHO regions, countries, publication years, continents, sample size, and diagnostic methods. A forest plot diagram was created to display the pooled prevalence with 95 % CIs. The funnel plot was used to assess publication bias in the analysis. Heterogeneity across studies was evaluated using the I2 index. Values below 25 %, 25–50 %, and over 50 % were categorized as low, moderate, and high heterogeneity. Furthermore, a sensitivity analysis was conducted to examine changes in the final weighted prevalence of *Blastocystis* spp. by excluding specific studies.

## 3 Results

### 3.1 Article selection

**Fig. 1:**
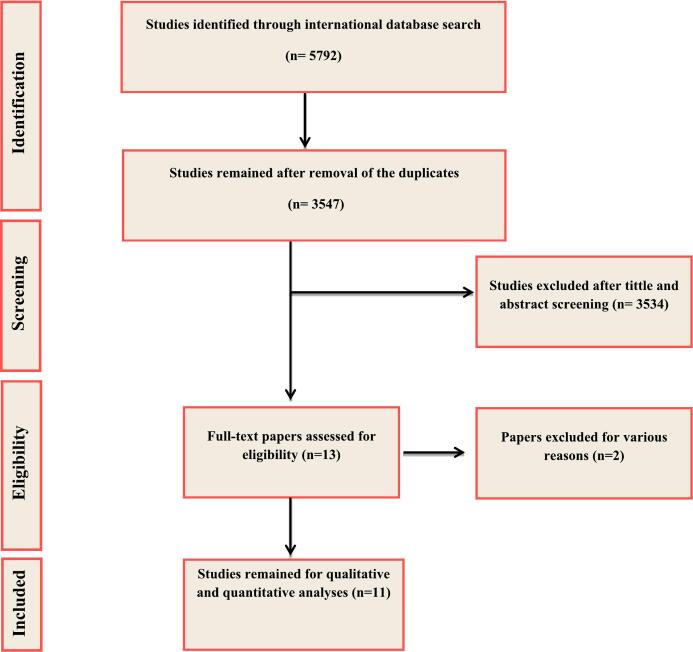
Flowchart of the included eligible studies in the present study.

Rigorous searches of four international databases found a total of 5792 initial records. After removing duplicates and reviewing the remaining 3547 records, a total of 13 articles were ultimately selected. Additionally, a quality assessment based on JBI criteria led to the exclusion of two more studies. Finally, a total of 11 relevant papers (11 datasets) met the inclusion criteria for this study (Fig. 1).

### 3.2 Qualitative and quantitative characteristics of the papers included

The analysis included 11 articles/datasets (five related to fish, five to bivalves, and one to sponges) published between 1997 and 2024. Out of the 1329 marine hosts, 742 were fish, 567 were bivalves, and 20 were sponges. France and Mexico published the most studies with two papers each, followed by single papers from each of Chile, China, Germany, Iran, Malaysia, Poland, and Qatar. Sample sizes ranged from 18 to 374 marine animals in the 11 studies. Seven papers detailed the subtype distribution of *Blastocystis* spp. in marine animals. Molecular method was the prevalent diagnostic technique in eight studies, with microscopy and culture techniques utilized in one and two studies, respectively (Table 1). The evaluation with the JBI checklist indicated that 6 papers were classified as high quality (>6 points), while the other 5 articles were deemed to be of moderate quality (4–6 points) (Supplementary Table 1).

**Table 1:** The main data from 11 studies in this review about the prevalence and subtype distribution of *Blastocystis* spp. in marine animals.

Author, year	Examined animals	Most infected animals	Time tested	Country	Total no.	Infected no.	Prevalence (%)	Method	STs e
Konig and Müller, 1997	Fish	**UC**^a^	**UC**	**Germany**	18	**2**	**11.1**	Cl b	**–**
Słodkowicz-Kowalska et al., 2015	Mussels	**UC**	**2012**	**Poland**	114	**4**	**3.5**	Mic c	**–**
Martínez-Barbabosa et al., 2018	Oysters	**UC**	**UC**	**Mexico**	30	**15**	**50**	Mic and Mol d	**–**
Compean et al., 2018	Oysters	**UC**	**2016–2017**	**Mexico**	250	**166**	**66.4**	Mic and Mol	**ST1 and UN**^f^
Gantois et al., 2020	Fish	**Herring**	**2019**	**France**	374	**16**	**4.3**	Mol	**ST8, ST10**^g^**, ST7, ST2, ST4, ST3**
Rauff-Adedotun et al., 2022	Fish	**UC**	**UC**	**Malaysia**	123	**0**	**0**	Cl	**–**
Wang et al., 2024	Fish	**Chinese sturgeon**	**2020–2022**	**China**	27	**3**	**11.1**	Mol	**ST1 and UN**
Ryckman et al., 2024	Mussels	**UC**	**2023**	**France**	100	**62**	**62**	Mol	**ST3, ST44, ST14, ST26, ST7, ST23, Mixed/UN**
Suarez et al., 2024	Mussels	**UC**	**2022**	**Chile**	73	**9**	**12.3**	Mol	**ST3 and UN**
Boughattas et al., 2024	Sponges	**UC**	**UC**	**Qatar**	20	**2**	**10**	Mol	**ST3**
Asghari et al., 2024a	Fish	**Narrow-barred mackerel**	**2022–2023**	**Iran**	200	**4**	**2**	Mol	**ST2, ST1, ST7**

a Unclear.

b Culture method.

c Microscopic detection.

d Molecular detection.

e Subtypes.

f Unidentified subtypes.

g This subtype, identified as ST10, exhibits a sequence similarity of 92 % with sequences found in GenBank, suggesting that it may be a new subtype.

### 3.3 Overall prevalence of *Blastocystis* spp. in marine animals

**Fig. 2:**
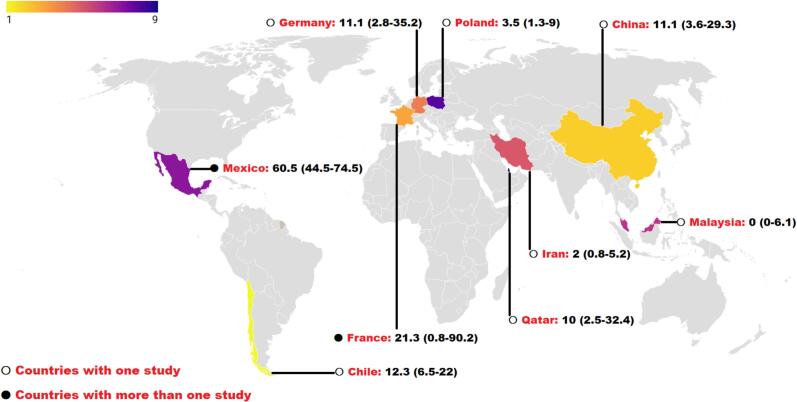
The global occurrence of *Blastocystis* spp. in marine animals across different countries (the numbers following the country names and the cases in parentheses indicate the prevalence rate of *Blastocystis* spp.)

**Fig. 3:**
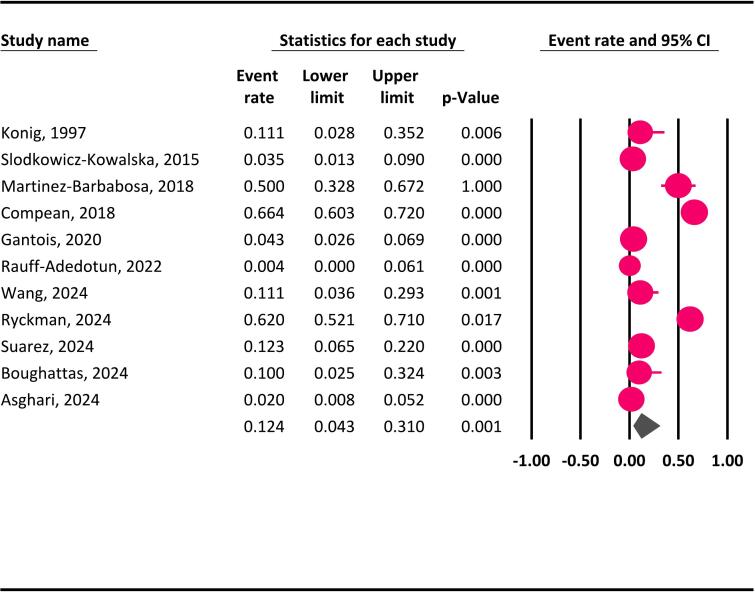
The worldwide prevalence of *Blastocystis* spp. in marine animals using a random-effects model and 95 % CIs.

The global occurrence of *Blastocystis* spp. in marine animals across different countries is illustrated in Fig. 2. This study revealed that 12.4 % (95 % CI: 4.3–31 %) of global marine animals were infected by *Blastocystis* spp. (Fig. 3). Statistical analysis revealed a considerable degree of heterogeneity among the included studies in the current systematic review and meta-analysis (Q = 328.1, *I*^*2*^ = 96.9 %, *P* = 0.000).

### 3.4 Pooled prevalence of *Blastocystis* spp. based on marine animal types

Among marine animals, bivalves exhibited the highest *Blastocystis* spp. infection rate at 32 % (95 % CI: 13–59.7 %), followed by sponges with a single study at 10 % (95 % CI: 2.5–32.4 %), and fish at 4.4 % (95 % CI: 2–9.3 %) (Table 2 and Supplementary Fig. 1).

**Table 2:** Subgroup analysis of *Blastocystis* spp. in marine animals by publication year, continent, WHO region, country, sample size, and diagnostic method.

Subgroup variable	Prevalence % (95 % CI)	Heterogeneity (Q)	df (Q)	I^2^ (%)	P-value
**Publication year**					
<2020	24.8 (5.5–65.3)	69.2	3	95.7	*P* < 0.05
2020–2024	7.8 (1.8–28.8)	166.4	6	96.4	P < 0.05
**Continent**					
Asia	4.1 (1.2–13.4)	9.6	3	68.7	P < 0.05
Europe	12.1 (1.4–57.6)	141.6	3	97.9	P < 0.05
North America	60.5 (44.5–74.5)	3.1	1	67.4	*P* > 0.05
South America	12.3 (6.5–22)	0	0	0	P > 0.05
**WHO region**					
AMR	40 (12.2–76.2)	49.1	2	95.8	P < 0.05
EMR	4.2 (0.8–18.5)	3.5	1	71.8	P > 0.05
EUR	12.1 (1.4–57.6)	141.6	3	97.9	P < 0.05
WPR	2.8 (0.1–44.3)	4.9	1	79.7	P > 0.05
**Country**					
Chile	12.3 (6.5–22)	0	0	0	P > 0.05
China	11.1 (3.6–29.3)	0	0	0	P > 0.05
France	21.3 (0.8–90.2)	120.1	1	99.2	P < 0.05
Germany	11.1 (2.8–35.2)	0	0	0	P > 0.05
Iran	2 (0.8–5.2)	0	0	0	P > 0.05
Malaysia	0 (0–6.1)	0	0	0	P > 0.05
Mexico	60.5 (44.5–74.5)	3.1	1	67.4	P > 0.05
Poland	3.5 (1.3–9)	0	0	0	P > 0.05
Qatar	10 (2.5–32.4)	0	0	0	P > 0.05
**Sample size**					
<100	17 (6.8–36.3)	20.3	4	80.3	P < 0.05
100–400	9.5 (1.8–37.5)	288.2	5	98.3	P < 0.05
**Diagnostic method**					
Cl	2.7 (0.1–43.8)	4.6	1	78.1	P < 0.05
Mic	3.5 (1.3–9)	0	0	0	P > 0.05
Mol	18.7 (6–45.2)	277.1	7	97.5	P < 0.05

### 3.5 Subtype distribution of *Blastocystis* spp. in marine animals

The findings revealed that marine animals can harbor different *Blastocystis* spp. STs (ST1-ST4, ST7, ST8, ST10, ST14, ST23, ST26, and ST44), with all types, except the last two, capable of spreading to humans. In particular, fish were identified as carriers of ST1-ST4, ST7, ST8, ST10, whereas bivalves carried ST1, ST3, ST44, ST14, ST26, ST7, and ST23 (Table 1).

### 3.6 Weighted prevalence of *Blastocystis* spp. in marine animals based on examined subgroups

The subgroup-based prevalence of *Blastocystis* spp. in marine animals is displayed in Table 2 and Supplementary Figs. 2–7.

### 3.7 Sensitivity analysis

Based on the sensitivity analysis, excluding particular datasets on *Blastocystis* spp. in marine animals did not notably alter the overall frequency (Supplementary Fig. 8).

### 3.8 Publication bias

**Fig. 4:**
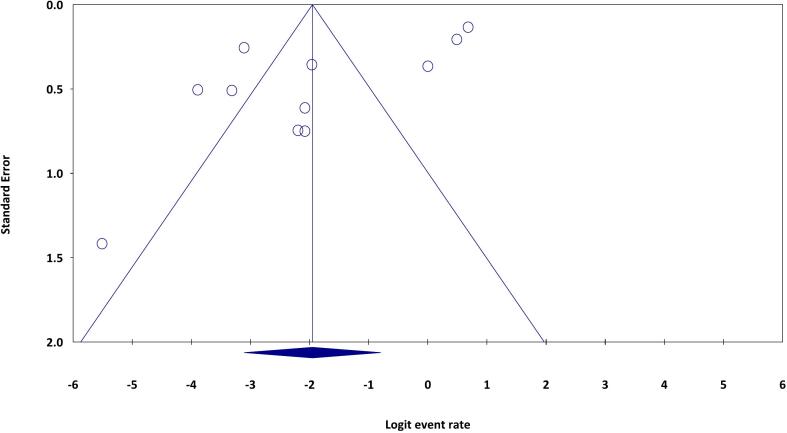
The funnel plot shows the publication bias in the present study.

A significant publication bias was detected in the current systematic review and meta-analysis (Egger's regression: intercept = − 6.805, 95 % lower limit = − 12.12, 95 % upper limit = − 1.48, t-value = 2.89, *P* = 0.017) (Fig. 4).

## 4 Discussion

The present review with 12.4 % (95 % CI: 4.3–31 %) found a moderate occurrence of *Blastocystis* spp. in marine animals, with bivalves showing a higher occurrence (32 %; 95 % CI: 13–59.7 %) compared to sponges (10 %; 95 % CI: 2.5–32.4 %) and fish (4.4 %; 95 % CI: 2–9.3 %). Of note, due to limited research, the true prevalence of *Blastocystis* spp. in these hosts remains unclear. Therefore, caution is advised when interpreting the prevalence rates from this study's analysis. Of note, in areas with high marine animal consumption, the risk of parasitic infections such as *Blastocystis* spp. should be taken seriously. After conducting the sensitivity analysis, the exclusion of specific datasets on *Blastocystis* spp. in marine animals did not significantly change the overall frequency. Furthermore, following the elimination of these datasets, the estimated prevalence of *Blastocystis* spp. in marine animals was around 9.6–15.3 %.

Subgroup analysis by year of publication revealed that more recent studies indicate a lower prevalence [7.8 % (95 % CI 1.8–28.8)] of *Blastocystis* spp. in marine animals. This shift could be attributed to the adoption of advanced molecular techniques and more accurate reporting of infection rates. Furthermore, research conducted across WHO regions, continents, and countries revealed a significantly high prevalence of *Blastocystis* spp. infection in marine animals in the AMR region [40 % (95 % CI: 12.2–76.2)] and on the continent of North America including Mexico [60.5 % (95 % CI: 44.5–74.5)]. Adherence to health protocols and raising public awareness regarding the consumption of raw or undercooked food in these regions is strongly advised. Increasing the sample size from <100 to 100–400 resulted in nearly halving the reported prevalence of *Blastocystis* spp. in marine animals, underscoring the significance of a large sample size in epidemiological research to ascertain the accurate prevalence of the targeted infection. Molecular methods (18.7 %; 95 % CI: 6–45.2) showed notably better diagnostic accuracy for identifying *Blastocystis* spp. in samples from marine animals compared to microscopic (3.5 %; 95 % CI: 1.3–9) and culture (2.7 %; 95 % CI: 0.1–43.8) methods. However, the number of studies varied significantly across diagnostic methods, and with an equal number of studies, the results could change entirely. Overall, the analyses in this study are mainly derived from a small pool of research and necessitate thorough interpretation.

Animals play a crucial role in the One-Health policy related to human health (Murtaugh et al., 2017). Out of the 40–44 reported STs of *Blastocystis* spp., 17 are zoonotic (ST1-ST10, ST12-ST14, ST16, ST23, ST35, and ST41) (Santin et al., 2024). This review found that marine animals can carry various *Blastocystis* spp. STs (ST1-ST4, ST7, ST8, ST10, ST14, ST23, ST26, and ST44), many of which (ST1-ST4, ST7, ST8, ST10, ST14, and ST23) can be transmitted to humans. Fish and bivalves were found to carry four STs (ST1-ST4, ST7, ST8, and ST10) and (ST1, ST3, ST44, ST14, ST26, ST7, and ST23), respectively. Overall, the research suggests that consuming raw or undercooked marine animals can lead to the spread of zoonotic *Blastocystis* spp. STs, potentially causing digestive issues. Furthermore, these animals can be consumed by larger predators, expanding the transmission cycle of *Blastocystis* spp., necessitating control and preventive actions in this area.

Parasitic infections like *Blastocystis* spp. can be transmitted from marine animals to humans through various means, including consumption of contaminated seafood, contact with infected water, environmental contamination, and food preparation practices. Preventive measures to reduce transmission include thoroughly cooking seafood, practising good hygiene, avoiding contaminated water, ensuring safe food preparation, and undergoing regular health check-ups. Following these practices can significantly lower the risk of transmission. To enhance the sampling of marine animals for studying parasitic infections like *Blastocystis* spp., future research can benefit from strategies such as collaborating with fisheries, employing non-invasive techniques like collecting water or fecal samples, and utilizing technologies like drones or underwater robots for preliminary surveys to locate and identify target species without disturbance.

This review encountered certain constraints, including a scarcity of prevalence data in many global regions, imprecise diagnostics such as molecular tests with good sensitivity and specificity, the inclusion of only one study in certain marine animal categories (e.g. sponges), and reliance on studies with limited sample sizes. These limitations hindered arriving at a definitive conclusion, yet our findings provided an assessment of the present status of *Blastocystis* spp. in marine animals.

## 5 Conclusion

This systematic review and meta-analysis study presented a 12.4 % moderate prevalence of *Blastocystis* spp. in marine animals, indicating that various species like fish, bivalves, and sponges can be infected with this protozoan parasite. Our findings highlighted that marine animals serve as proper hosts for different *Blastocystis* spp. STs, including zoonotic ones. Therefore, the significance of *Blastocystis* spp. infection, particularly its zoonotic STs, in the transmission from marine animals to humans and marine predatory species, should not be underestimated. Overall, investigating the prevalence and STs distribution of *Blastocystis* spp. in marine animals is an emerging field that warrants further exploration.

## CRediT authorship contribution statement

**Mohammad Ghafari-Cherati:** Methodology, Investigation. **Amin Karampour:** Methodology, Investigation. **Seyedeh-Sara Nazem-Sadati:** Methodology, Investigation. **Ali Asghari:** Writing – review & editing, Writing – original draft, Methodology, Investigation, Conceptualization.

## Declaration of competing interest

The authors declare no potential conflicts of interest with respect to the research, authorship, and/or publication of this article.

## Appendix A Supplementary data

Supplementary material 1 Image 1

Supplementary material 2 Image 2

Supplementary material 3 Image 3

Supplementary material 4 Image 4

Supplementary material 5 Image 5

Supplementary material 6 Image 6

Supplementary material 7 Image 7

Supplementary material 8 Image 8

Supplementary material 9 Image 9
