# Supplementary material for: Foodborne concerns of Blastocystis spp. in marine animals (fish, bivalves, and sponges): A systematic review and meta-analysis of global prevalence and subtypes distribution
Source: Food Waterborne Parasitol. 2024 Aug 25;36:e00242. doi: 10.1016/j.fawpar.2024.e00242 (PMC11399649; doi:10.1016/j.fawpar.2024.e00242)
Supplement: Supplementary file 1 — Supplementary material 1 [file mmc1.docx]

**Supplementary Fig. 1.** The global prevalence of *Blastocystis* spp. in marine animals based on animal type.
