# Supplementary material for: Foodborne concerns of Blastocystis spp. in marine animals (fish, bivalves, and sponges): A systematic review and meta-analysis of global prevalence and subtypes distribution
Source: Food Waterborne Parasitol. 2024 Aug 25;36:e00242. doi: 10.1016/j.fawpar.2024.e00242 (PMC11399649; doi:10.1016/j.fawpar.2024.e00242)
Supplement: Supplementary file 9 — Supplementary material 9 [file mmc9.docx]

**Supplementary Table 1**

**JBI critical appraisal checklist applied for included studies**

| Author Name/Year | Sample was representative? | Participants appropriately recruited? | Sample size was adequate? | Study subjects and the setting described? | Data analysis conducted | Objective, standard criteria, reliably used? | Appropriate statistical analysis used | Confounding factors/ subgroups/ differences identified and accounted? | Subpopulations identified using objective criteria | Overall quality |
| --- | --- | --- | --- | --- | --- | --- | --- | --- | --- | --- |
| Konig, 1997 | Yes | Yes | Yes | Yes | Yes | Yes | No | No | No | 4/9 |
| Slodkowicz-Kowalska, 2015 | Yes | No | Yes | Yes | No | Yes | No | No | No | 6/9 |
| Martinez-Barbabosa, 2018 | Yes | No | Yes | No | Yes | No | Yes | No | No | 4/9 |
| Compean, 2018 | Yes | Yes | No | Yes | No | Yes | No | Yes | No | 5/9 |
| Gantois, 2020 | Yes | Yes | Yes | Yes | Yes | No | Yes | Yes | No | 7/9 |
| Rauff-Adedotun, 2022 | Yes | Yes | Yes | Yes | Yes | No | Yes | No | No | 6/9 |
| Wang, 2024 | Yes | Yes | Yes | Yes | Yes | No | Yes | Yes | Yes | 8/9 |
| Ryckman, 2024 | Yes | Yes | Yes | Yes | Yes | Yes | Yes | Yes | No | 8/9 |
| Suarez, 2024 | Yes | Yes | Yes | Yes | Yes | No | Yes | No | Yes | 7/9 |
| Boughattas, 2024 | Yes | Yes | Yes | Yes | Yes | No | Yes | Yes | Yes | 8/9 |
| Asghari, 2024 | Yes | No | Yes | Yes | Yes | Yes | Yes | No | Yes | 7/9 |
